# Pre- and postoperative pain management practices in fracture surgery: A bicentric prospective observational study in Ethiopia

**DOI:** 10.1007/s00423-025-03837-y

**Published:** 2025-08-13

**Authors:** Mestawet Getachew, Anners Lerdal, Tsegaye Melaku, Maren Falch Lindberg

**Affiliations:** 1 Department of Clinical Pharmacy, School of Pharmacy, Institute of Health, Jimma University, Jimma, Ethiopia; 2 Research Department, Lovisenberg Diaconal Hospital, Oslo, Norway; 3 Department of Public Health and Interdisciplinary Health Sciences, Institute of Health and Society, Faculty of Medicine, University of Oslo, Oslo, Norway; 4 Department of Surgery, Lovisenberg Diaconal Hospital, Oslo, Norway

**Keywords:** Pain management, Orthopedic surgery, Pain management index, Traumatic fractures, Analgesics

## Abstract

**Background:**

Acute pain is common following orthopedic trauma and surgery. This study aims to evaluate the adequacy of pre- and postoperative pain management in traumatic fracture patients undergoing orthopedic surgery in Ethiopia.

**Methods:**

A prospective cohort study was conducted at two Ethiopian trauma centers from January 2019 to October 2021. Preoperatively, data was obtained on sociodemographic factors, substance use, type of injury, pain and psychological factors. Pain was assessed again 24 h following surgery. Pain management adequacy was evaluated using the Pain Management Index (PMI), based on the World Health Organization’s pain treatment framework. The PMI was determined by subtracting the patient’s pain intensity from the strength of the prescribed analgesic scores range from − 3 to + 3. Negative values indicate inadequate pain control.

**Results:**

Of the 220 patients enrolled, 218 completed the study. Preoperative pain was inadequately managed in 74.8% of patients, improving to 42.2% postoperatively. Most patients reported mild (23.3%), moderate (43.1%), or severe (30.8%) pain preoperatively, yet 56.4% received no analgesics. At 24 h post-surgery, the patients reported mild (5.0%), moderate (53.7%), and severe (41.3%) pain, with 99.1% receiving analgesics. Notably, no patients with severe pain were given strong opioids at any time point. Lower educational level was associated with inadequate preoperative pain management (AOR: 3.18; 95% CI: 1.19–8.54). Alcohol use (AOR: 2.80; 95% CI: 1.30–6.05), higher anxiety (AOR: 1.17; 95% CI: 1.05–1.30), and higher depression scores (AOR: 0.77; 95% CI: 0.68–0.88) were associated with inadequate pain management 24 h post-surgery.

**Conclusions:**

Most patients with traumatic fractures received inadequate perioperative pain management, especially before surgery. Strong opioids were not used even in cases of severe pain. Socio-demographic and psychological factors were significantly associated with inadequate pain management.

## Background

Orthopedic injuries, particularly fractures, represent a significant global public health burden [1] and remain a critical challenge in Ethiopia [2]. Pain is a common and highly distressing symptom among individuals with orthopedic injuries [3, 4]. Moreover, orthopedic surgeries are among the most painful procedures, often resulting in some of the highest reported pain intensity scores compared to other surgical interventions [5]. Consequently, effective pain management is a fundamental component of orthopedic care [4, 6]. Inadequate pain control can lead to a range of adverse outcomes, including increased complications, persistent pain, impaired physical function manifested as limited mobility and difficulty performing daily activities, delayed recovery, prolonged opioid use, diminished quality of life, and rising healthcare costs [7, 8].

Despite the availability of effective treatments, many patients undergoing orthopedic surgery continue to experience unrelieved pain. Studies from high-income countries report that 20–56% of patients experience moderate to severe pain during the early postoperative period [9–12]. Although research on orthopedic patients in Ethiopia is limited, one study found that 70.5% of patients experienced moderate to severe postoperative pain, highlighting a significant gap in pain management practices [13]. Additional evidence from Ethiopia indicates that nearly half of trauma patients treated in emergency settings did not receive adequate analgesia [14], underscoring a critical deficiency in the timely and effective delivery of pain relief. Similarly, a study from Nepal found that 61.5% of orthopedic surgery patients received inadequate analgesia during the immediate postoperative period [15]. These findings point to a persistent and widespread challenge in achieving effective pain management, across both emergency and surgical care settings, particularly in low- and middle-income countries where systemic limitations may hinder optimal analgesic practices.

Recent literature emphasizes the importance of a multimodal approach to pain management for patients with fractures and those undergoing orthopedic surgery. This strategy integrates non-opioid medications such as NSAIDs and acetaminophen, with non-pharmacological interventions like rest, ice, and early mobilization, along with psychological support to manage the emotional impact of pain. Opioids should be reserved for cases where other modalities are insufficient, and when used, they should be prescribed at the lowest effective dose for the shortest possible duration, preferably in immediate-release formulations to minimize risk. Additionally, regional anesthesia techniques, including nerve blocks, offer targeted pain relief and reduce reliance on systemic opioids. Collectively, these strategies enhance pain control, accelerate rehabilitation, improve patient outcomes, and reduce adverse effects [4, 16, 17].

Despite advancements in pain management, there is a notable lack of research on the adequacy of pain control among patients with traumatic fractures in Ethiopia, particularly during both the preoperative and postoperative periods. Existing literature is largely limited to a single study focusing on emergency care settings [14] leaving a significant gap in understanding pain management throughout the surgical continuum. This study aims to assess the adequacy of analgesic use before surgery and 24 h postoperatively in patients with traumatic fractures undergoing orthopedic procedures, and to identify factors associated with analgesic adequacy at each stage.

## Methods

### Study design, setting, and period

This prospective observational cohort study was conducted at the trauma centers of two University teaching Hospitals in Ethiopia: Jimma Medical Center (JMC) and Addis Ababa Burn Emergency and Trauma (AaBET) Hospital. Both centers provide important orthopedic care, including fracture management. However, standardized protocols for preoperative fracture management are largely absent at the two settings. The study period spanned from January 2019 to October 2021.

### Participants and perioperative data collection

Prior to data collection, the study protocol was reviewed and approved by the relevant research ethics boards: the Institutional Review Boards of Jimma University (JHRPGD/510/2018), St. Paul’s Hospital Millennium Medical College (PM23/406), and the Regional Committee for Medical Research Ethics - South-East Norway (2017/1609/REK).

Data were collected concurrently at both trauma centers throughout the study period. Trained research assistants, independent of the clinical care teams, were responsible for data acquisition. On the day before surgery, the research assistants screened the surgical waiting list to identify eligible patients based on the following inclusion criteria: age 18 years or older; presence of upper and/or lower extremity fractures due to trauma; scheduled for elective orthopedic surgery at one of the study sites; fully conscious and without cognitive impairment. Eligible patients were approached by the research assistants and provided with detailed information about the study objectives and their expected role. Written informed consent was obtained from all participants. For participants who were illiterate (16.4%), informed consent was obtained orally in the presence of a witness, who also provided a signature to confirm the participant’s agreement.

Data collection followed a structured approach, combining comprehensive chart reviews with patient interviews using standardized questionnaires. Preoperative data included demographic variables (age in years, sex, residence, education level, employment status), lifestyle factors (tobacco use, alcohol consumption, khat use), type of injury, and psychological factors (anxiety, depression, pain catastrophizing).

Intraoperative data were extracted from surgical and anesthetic records and included the type of anesthesia administered and duration of surgery (in hours). Pain intensity at the fracture site was assessed on the day prior to surgery and again 24 h postoperatively. Analgesic use was assessed through review of medical records.

### Measurements

Anxiety and depression were assessed using The Hospital Anxiety and Depression Scale (HADS). This 14-item instrument comprise two subscales; one for anxiety and one for depression, each consisting of seven items assessing symptoms of anxiety and depression throughout the past week. Responses are rated on a 4-point Likert scale ranging from 0–3 scored between “not at all” and “most of the time,” yielding two sub-scores ranging from 0 to 21. Higher scores indicate more severe symptoms of anxiety or depression. For this study, a validated Ethiopian Version of the HADS was used [18, 19].

The Pain Catastrophizing Scale (PCS) was used to measure patients’ level of pain catastrophizing. Originally developed by Sullivan et al. in 1995, the PCS consist of 13 items rated on a 5-point Likert from 0 “not at all” to 4 “all the time” resulting in a total score ranging from 0 to 52. Higher scores indicate higher levels of catastrophizing [20]. As no validated Ethiopian version was available at the time of this study, the PCS was translated and culturally adapted into Amharic and Afan Oromo - the two most often spoken languages in the study settings - using a 5-step procedure recommended for translating and cross-cultural adaptation of health measurement tools [21].

Pain intensity was measured using the Brief Pain Inventory (BPI), which has been validated for use in Ethiopia [22]. In this study, the patient’s worst pain intensity was measured using an 11-point Numeric Rating Scale (NRS) where 0 indicates “no pain” and 10 indicates “worst imaginable pain”.

The primary outcomes of this study were the prevalence of inadequate pain management at two time points: prior to surgery and 24 h post-surgery, using the Pain Management Index (PMI). The PMI was calculated in accordance with the World Health Organization (WHO) guidelines [23] by subtracting the patients’ pain intensity from the potency level of the analgesic administered. Pain intensity was determined using the worst pain score from the BPI 11-point NRS, categorized as follows: 0 = no pain, 1 = mild (1–3), 2 = moderate (4–7), and 3 = severe (8–10). Analgesics were classified by potency as follows: 0 (no analgesic), 1 (non-opioid analgesics: e.g., acetaminophen or nonsteroidal anti-inflammatory drugs {NSAIDs}), 2 (weak opioids: e.g., codeine, hydrocodone, tramadol), and 3 (strong opioids: e.g., morphine, methadone, oxycodone, hydromorphone, and fentanyl). The PMI score was derived by subtracting the pain severity category from the analgesic potency level resulting in a range from − 3 (a patient with severe pain receiving no analgesia) to + 3 (a patient receiving strong opioids without reporting pain). A negative PMI score reflects inadequate pain management, while a score of zero or above is considered indicative of adequate pain management [23, 24].

### Statistical analysis

All statistical analyses were conducted using IBM Statistical Package for Social Science (SPSS) version 25.0. Descriptive statistics were used to summarize the sociodemographic and clinical characteristics of the study participants. Categorical variables are presented as frequencies and percentages, while continuous variables are reported as means and standard deviations. Prior to performing bivariate and multivariate logistic regression analyses, a correlation analysis was conducted to examine potential multicollinearity among the covariates. The analysis of factors associated with inadequate pain management was carried out in two stages. First, bivariate logistic regression analyses were conducted for each independent variable including sociodemographic, surgical, psychological factors against the outcome variable PMI. Variables with a *p-value* < 0.25 in the bivariate analysis were retained for inclusion in multivariate logistic regression models. To assess the robustness of the multivariate logistic regression models, the Hosmer–Lemeshow goodness-of-fit test was applied. A non-significant result (*p* > 0.05) was interpreted as an indication of good model fit between the observed and predicted outcomes. Multicollinearity was evaluated using variance inflation factors (VIF); all predictors included in the final models had VIF values below 10 and tolerance above 0.1, which were considered acceptable. Results are reported as odds ratios (OR) with corresponding 95% confidence intervals and *p-values*, with statistical significance set at *p* < 0.05.

## Results

A total of 220 eligible patients consented to participate and completed the baseline assessment. Two patients were discharged against medical advice on the day of surgery and were therefore excluded from the analysis. The final sample comprised 218 patients who underwent elective surgery for traumatic fractures. Of these, 115 were recruited from JMC and 103 from AeBET Hospital yielding a retention rate of 99.1%.

### Sociodemographic, lifestyle, clinical, psychological, and surgical characteristics

The mean age of participants was 33.4 years (SD = 11.6). The majority were male (*n* = 176, 80.7%), and just over half (*n* = 112, 51.4%) resided in urban areas. Detailed information on participants’ sociodemographic characteristics, lifestyle habits, psychological profiles, and surgical-related factors is presented in Table 1.

**Table 1:** Sociodemographic, lifestyle, psychological, and surgical characteristics (*N* = 218)

Characteristic	Category	*n* (%)
Sex	Female	42 (19.3)
	Male	176 (80.7)
Education Level	No formal education	34 (16.4)
	Primary	88 (40.0)
	Secondary	65 (29.5)
	Diploma and degree	31 (14.1)
Residence	Urban	112 (51.4)
	Rural	106 (48.6)
Lifestyle	Smoker	25 (11.5)
	Alcohol use	102 (46.8)
	Khat Chewer	94 (43.1)
Injury mechanism	Road traffic injury	114 (52.5)
	Machine/conflict-related injury	52 (24.0)
	Falls	51 (23.5)
Injury location	Upper extremities	86 (39.4)
	Lower extremities	122 (56.0)
	Both	10 (4.5)
Type of anesthesia	General	79 (36.7)
	Spinal	94 (43.7)
	Nerve block	42 (19.5)
Duration of surgery (minutes) [Mean (SD)]		122.4 (57.9)
Psychological factors [Mean (SD)]	^£^Anxiety (HADS-A)	8.5 (4.3)
	^£^Depression (HADS-D)	11.9 (3.6)
	^®^Pain catastrophizing (PCS)	25.8 (8.5)

^**®**^ measured by the PCS, pain catastrophizing scale total scores with ranges of 0–52; ^£^measured by HADS, Hospital Anxiety and Depression scale. Anxiety HADS-A range of 0–21 and depression HADS-D range of 0–21; SD, standard deviations

###  Pain management practices prior to surgery and 24 h post-surgery

Prior to surgery, 6 (2.8%) patients reported no pain, 51 (23.3%) reported mild pain, 94 (43.1%) reported moderate pain, and 67 (30.8%) reported severe pain. Among those reporting any level of pain, 123 patients (56.4%) did not receive or take any form of analgesia prior to surgery. This group included 42 patients with mild pain, 44 with moderate pain, and 37 with severe pain. Among those who did receive analgesics, 17 patients (7.7%) were administered diclofenac, 61 (27.7%) received tramadol, and 11 (5.0%) received a combination of diclofenac and tramadol.

**Fig. 1:**
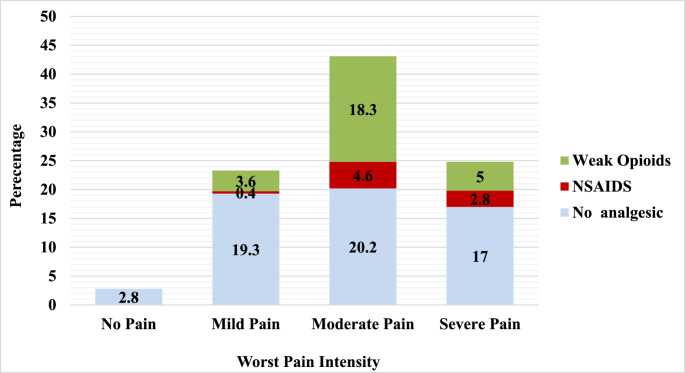
Analgesics use pattern among participants by worst pain intensity, prior to surgery. No pain (*n*=6); Mild Pain (*n*=51); Moderate Pain (*n*=94); Severe Pain (*n*=67)

**Fig. 2:**
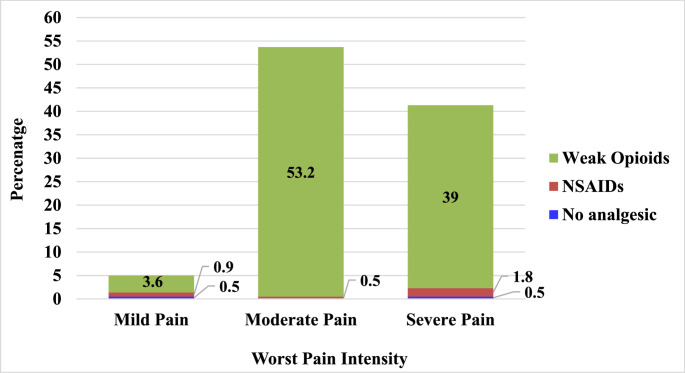
Analgesics use pattern of participants by worst pain intensity, 24 h post-surgery. Mild Pain (*n*=11); Moderate Pain (*n*=117); Severe Pain (*n*=90)

At 24 h post-surgery, almost all patients (*n* = 216, 99.1%) had received analgesic treatment. Of these, 11 patients (5.0%) reported mild pain, 117 (53.7%) reported moderate pain, and 90 (41.3%) reported severe pain. The analgesic regimens administered postoperatively included diclofenac alone in 7 patients (3.2%), tramadol alone in 138 (62.7%), a combination of tramadol and diclofenac in 69 (31.4%), tramadol with paracetamol in 1 patient (0.5%), tramadol with pethidine in 1 patient (0.5%). Despite the high proportion of patients reporting severe pain both preoperatively and post-surgery, none received strong opioids at either time points as shown in Figs. 1 or 2.

### Adequacy of pain management prior to surgery and 24 h post-surgery

**Fig. 3:**
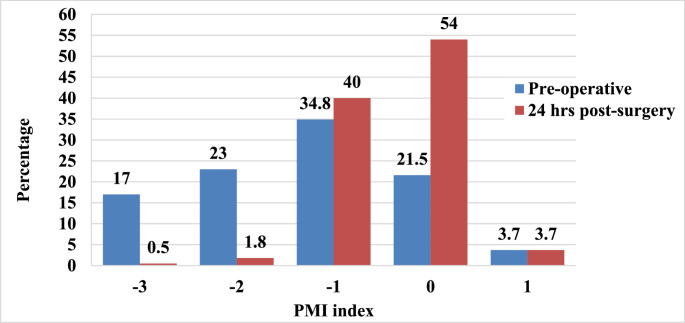
Pain management index (PMI) among participants prior to surgery and 24 h post-surgery. PMI index ≥ 0 = adequate pain management; PMI index < 0 = inadequate pain management (*N* = 218)

The adequacy of pain management assessed with the PMI is shown in Fig. 3. Prior to surgery, 163 patients (74.8%) had a negative PMI score, indicating inadequate pain management according to the WHO guidelines. At 24 h postoperatively, the number of patients with a negative PMI decreased to 92 (42.3%). A comparison of PMI before and after surgery was conducted using the Chi-Square test, which revealed a statistically significant improvement in PMI (*p* < 0.001). This indicates that the proportion of patients receiving inadequate pain management declined substantially from the preoperative to the postoperative period.

### Factors associated with inadequate pain management prior to surgery

Results from bivariate and multivariate logistic regressions analyses are presented in Table 2. In the bivariate analysis, lower level of education, urban residence, and upper extremity injuries were significantly associated with inadequate pain management prior to surgery. However, in the multivariate logistic regression, only lower level of education remained a significant predictor. Those with lower level of education had 3.2 times higher odds of receiving inadequate pain management compared to those with higher level of education (AOR = 3.18; 95% CI: 1.19–8.54; *p* = 0.022).

### Factors associated with inadequate pain management 24 h post-surgery

As shown in Table 3, the bivariate logistic regression model identified 9 factors associated with inadequate pain management post-surgery, including lower levels of education, urban residence, alcohol use, khat use, longer surgery duration, type of anesthesia, anxiety, depression and pain catastrophizing, have a significant association with inadequate pain management 24 h post-surgery. However, in the multivariate model, only three factors remained significant: alcohol use, anxiety, and depression.

Patients who used alcohol had 2.8 times higher odds of receiving inadequate pain management 24 h post-surgery compared to those who did not use alcohol (AOR = 2.80; 95% CI: 1.30–6.05; *p* = 0.008). Furthermore, each one-point increase in anxiety score was associated with a 17% increase in the odds of receiving inadequate pain management (AOR = 1.17; 95% CI: 1.05–1.30; *p* = 0.004). In contrast, each one-point increase in depression score was associated with a 23% reduction in the odds of receiving inadequate pain management (AOR = 0.77; 95% CI: 0.68–0.88; *p* < 0.001).

**Table 2:** Bivariate and multivariate analysis of factors associated with inadequate pain management prior to surgery among patients undergoing orthopedic surgery for traumatic fractures (*N* = 218)

Variables	Categories	Pain management Prior to surgery		Bivariate		Multivariate	
				COR (95%CI)	*P*-value	AOR (95%CI)	*P*-value
		Adequate (%)	Inadequate (%)				
Age (years) Mean **±** SD		33.4 **±** 11.6		0.99 (0.97–1.02)	0.496		
Sex	Female	9 (21.4)	33 (78.6)	1.29 (0.58–2.92)	0.529		
	Male	46 (26.1)	130 (73.9)	1			
Education Level	Primary	15 (17.0)	73 (83.0)	4.00 (1.63–9.85)	0.002*	3.18 (1.19–8.54)	0.022*
	Secondary	16 (24.6)	49 (75.4)	2.52 (1.02–6.23)	0.045*	2.14 (0.85–5.43)	0.108
	Diploma and degree	14 (45.2)	17 (54.8)	1		1	
Residence	Urban	35 (31.3)	77 (68.8)	0.51 (0.27–0.96)	0.037*	0.62 (0.28–1.39)	0.246
	Rural	20 (18.9)	86 (81.1)	1		1	
Smoking (Yes)		7 (28.0)	18 (72.0)	0.85 (0.34–2.16)	0.735		
Alcohol Use (Yes)		28 (27.5)	74 (72.5)	0.80 (0.44–1.48)	0.479		
Khat Use (Yes)		27 (28.7)	67 (71.3)	0.72 (0.39–1.34)	0.302		
Mechanism of injury	Road traffic accident	30 (26.3)	84 (73.7)	0.96 (0.45–2.04)	0.911		
	Machine/conflict-related injury	12 (23.1)	40 (76.9)	1.14 (0.46–2.80)	0.775		
	Falls	13 (25.5)	38 (74.5)	1			
Location of injury	Upper extremities	16 (18.6)	70 (81.4)	2.92 (0.74–11.56)	0.128*	4.75 (0.95–23.90)	0.058
	Lower extremities	35 (28.7)	87 (71.3)	1.66 (0.44–6.23)	0.455	3.53 (0.74–16.79)	0.113
	Both extremities	4 (40)	6 (60)	1		1	
^£^Anxiety (HADS-A) (Mean **±** SD)		8.5 ± 4.3		1.02 (0.95–1.09)	0.540		
^£^Depression (HADS-D) (Mean **±** SD)		11.9 ± 3.6		0.99 (0.90–1.07)	0.733		
^**®**^Pain catastrophizing (PCS) (Mean **±** SD)		25.8 ± 8.5		1.01(0.98–1.05)	0.534		

^§^ measured by the Brief Pain Inventory (BPI) “worst pain intensity” using numerical rating scale (NRS) and the pain intensity scores categorized as: (0) No pain, (1–3) mild pain, (4–7) moderate pain, and (8–10) severe pain

^**®**^ measured by the PCS, pain catastrophizing scale total score with a range of 0–52

^£^measured by HADS, Hospital Anxiety and Depression scale i.e. Anxiety HADS-A with range of 0–21 and depression HADS-D with a range of 0–21

SD, standard deviations; CI = confidence interval; *COR = crude odds ratio significant association at *p*-values < 0.25; *AOR = adjusted odds ratio significant association at *p*-values < 0.05

**Table 3:** Bivariate and multivariate analysis of factors associated with inadequate pain management 24 h post-surgery among patients undergoing orthopedic surgery for traumatic fractures (*N* = 218)

Variables	Categories	Pain management 24 h post-surgery		Bivariate		Multivariate	
		Adequate (%)	Inadequate (%)	COR (95%CI)	*P*-value	AOR (95%CI)	*P*-value
Age (years) Mean **±** SD		33.4 **±** 11.6		1.01(0.98–1.03)	0.550		
Sex	Female	9 (21.4)	33 (78.6)	0.81 (0.41–1.61)	0.549		
	Male	46 (26.1)	130 (73.9)	1			
Education Level	Primary	15 (17.0)	73 (83.0)	1.66 (0.71–3.87)	0.240*	2.97 (0.96–9.19)	0.059
	Secondary	16 (24.6)	49 (75.4)	1.21 (0.49–2.94)	0.671	2.03 (0.67–6.16)	0.211
	Diploma and degree	14 (45.2)	17 (54.8)	1		1	
Residence	Urban	35 (31.3)	77 (68.8)	0.73 (0.42–1.24)	0.242*	1.01 (0.46–2.19)	0.988
	Rural	20 (18.9)	86 (81.1)	1		1	
Smoking (Yes)		7 (28.0)	18 (72.0)	0.75 (0.31–1.77)	0.506		
Alcohol Use (Yes)		28 (27.5)	74 (72.5)	0.36 (0.21–0.64)	< 0.001*	2.80 (1.30–6.05)	0.008*
Khat Use (Yes)		27 (28.7)	67 (71.3)	0.59 (0.34–1.03)	0.066*	1.16 (0.54–2.46)	0.706
Mechanism of injury	Road traffic accident	30 (26.3)	84 (73.7)	1.35 (0.69–2.64)	0.385		
	Machine/conflict-related injury	12 (23.1)	40 (76.9)	0.82 (0.37–1.83)	0.629		
	Falls	13 (25.5)	38 (74.5)	1			
Location of injury	Upper extremities	16 (18.6)	70 (81.4)	0.89 (0.23–3.39)	0.863		
	Lower extremities	35 (28.7)	87 (71.3)	1.27 (0.34–4.74)	0.719		
	Both extremities	4 (40)	6 (60)	1			
Anesthesia type	General	17 (21.5)	62 (78.5)	2.20 (0.99–4.91)	0.054*	1.52 (0.49–4.65)	0.467
	Spinal	24 (25.5)	70 (74.5)	1.85 (0.85–4.06)	0.124*	1.26 (0.41–3.81)	0.688
	Nerve block	13 (31.0)	29 (69.0)	1		1	
Duration of surgery(minutes) Mean **±** SD		122.4 ± 57.9		1.01(1.00-1.01)	< 0.001*	1.01 (1.00-1.01)	0.046
^£^ Anxiety (HADS-A); Mean ± SD		8.5 ± 4.3		1.15 (1.07–1.23)	< 0.001*	1.17 (1.05–1.30)	0.004*
^£^ Depression (HADS-D); Mean ± SD		11.9 ± 3.6		0.89 (0.82–0.97)	0.007*	0.77 (0.68–0.88)	< 0.001*
^**®**^ Pain catastrophizing (PCS); Mean ± SD		25.8 ± 8.5		1.03 (1.00-1.07)	0.053*	1.04 (0.99–1.10)	0.127

^§^ measured by the Brief Pain Inventory (BPI) “worst pain intensity” using numerical rating scale (NRS) and the pain intensity scores categorized as: (0) No pain, (1–3) mild pain, (4–7) moderate pain, and (8–10) severe pain

^**®**^measured by the PCS, pain catastrophizing scale total score with a range of 0–52

^£^measured by HADS, Hospital Anxiety and Depression scale i.e. Anxiety HADS-A with a range of 0–21 and depression HADS-D with a range of 0–21; SD, standard deviations; CI = confidence interval; *COR = crude odds ratio significant association at *p-values* < 0.25; *AOR = adjusted odds ratio significant association at *p*-values < 0.05

## Discussion

This study revealed a high prevalence of inadequate pain management among patients undergoing orthopedic surgery for traumatic fractures in Ethiopia. Specifically, 74.8% of patients received inadequate analgesia prior to surgery, which improved to 42.3% at 24 h post-surgery. Despite this improvement, a substantial proportion of patients continued to experience moderate to severe pain, and notably, none received strong opioids at either time point.

More than half of the patients who reported pain prior to surgery did not receive any form of analgesia. This finding is consistent with from studies conducted in Nigeria [25] and Los Angeles [26], which also examined the administration of preoperative analgesics. However, it is important to note that the Los Angeles study included patients with head trauma, whereas the present study focused exclusively on fully conscious adult patients with traumatic fractures and no cognitive impairment. This difference in patient populations may partially explain the variation in analgesic practices across settings. Nevertheless, the findings underscore the urgent need to improve preoperative pain management, particularly in trauma care contexts.

According to our findings, patients received inadequate pain medication prior to surgery. Although the proportion of patients receiving inadequate pain management decreased significantly postoperatively, from 74.8 to 42.3%, a large number of patients still experienced insufficient pain relief. This finding is consistent with findings from Brazil [27] where postoperative analgesic adequacy was found to be higher than in the preoperative period, suggesting that preoperative pain management often receives less clinical attention than postoperative pain. Similar trends have been reported in other low- and middle-income countries (LMICs). For example, a study in Nepal reported that 61.5% of orthopedic surgery patients received inadequate analgesia in the immediate postoperative period [28], while research from Nigeria found that over 70% of trauma patients did not receive any form of analgesia prior to surgery [28]. Several barriers to effective pain management in LMICs in Africa have been identified in the literature [29], including limited clinician knowledge, negative provider attitudes, inadequate pain assessment, patients’ reluctance to report pain, misconceptions regarding pain and pain medications, resource constraints, and a shortage of essential analgesic drugs. This combination of individual, systemic and contextual barriers may help explain the high proportion of patients with inadequate pain management in our study. However, it is important to note that identifying and analyzing such barriers was beyond the scope of our study.

Despite the relatively high number of patients reporting severe pain both before and after surgery, none received strong opioids. This finding is likely multifactorial in origin. First, in LMICs such as Ethiopia, strong opioids including morphine and other potent analgesics are often unavailable or inconsistently supplied. Second, restrictive national policies aimed at preventing misuse and dependence may severely limit the clinical use of opioids, even in cases where they are medically indicated. Third, health care providers may be reluctant to prescribe strong opioids due to concerns about adverse effects as well as exaggerated fears of tolerance, dependence, and addiction [30]. However, the severity of pain is a critical determinant in selecting appropriate analgesic therapy, particularly in orthopedic care. Clinical guidelines generally recommend short-term use of strong opioids for hospitalized patients experiencing severe postoperative or trauma-related pain [31]. The absence of such treatment in this study highlights a potential mismatch between pain severity and analgesic choice suggesting inappropriate pain management for a significant proportion of patients. This issue warrants urgent attention from healthcare institutions and policy makers to ensure that pain management practices are both evidence-based and patient-centered.

Another key finding of this study was that patients with only primary level of education were three times more likely to receive inadequate pain management prior to surgery compared to those with higher educational attainment. This result is consistent with findings from a study conducted in Greece [32]. One possible explanation is that patients with lower education levels may feel less confident or less empowered to communicate their pain effectively, which can lead to underassessment by healthcare providers and, consequently, inadequate treatment. To address such disparities, clinicians should be attentive to patients’ educational backgrounds and tailor their communication and patient education strategies, accordingly, ensuring that all patients are adequately informed and encouraged to report their symptoms.

Additionally, this study found that patients who reported alcohol use were nearly three times more likely to receive inadequate pain management 24 h post-surgery compared to non-users. Existing literature suggest that while moderate alcohol consumption may be associated with improved pain outcomes, heavy or problematic drinking is linked to poorer pain control [33, 34]. However, the present study did not collect detailed data on the frequency, quantity or patterns of alcohol consumption. Therefore, it is possible that the observed association may be confounded by other unmeasured factors.

Preoperative anxiety is a common psychological response among surgical patients in low- and middle-income countries, affecting approximately one in two patients, according to Bedaso et al. [35]. Growing evidence suggest that preoperative psychological factors such as anxiety [36, 37] and depression [37–39] are associated with increased postoperative pain severity and higher analgesic consumption [36, 40]. In the present study, we identified a significant association between preoperative psychological status and the adequacy of pain management. Specifically, patients with higher anxiety scores were more likely to receive inadequate pain management 24 h after surgery. Conversely, and somewhat unexpectedly, those with higher depression scores were less likely to receive inadequate pain management. This inverse association between depressive symptoms and pain management inadequacy may be may be explained by the tendency of individuals with depression to exhibit reduced emotional reactivity and diminished motivation to report pain or seek additional support [41]. Such behavioral patterns may lead to underreporting pain and a passive acceptance of discomfort, which could influence both clinical assessment and treatment decisions. Given the limited body of research on this specific relationship, direct comparisons with other studies are challenging. Nonetheless, these findings underscore the importance of incorporating preoperative psychological assessments into routine surgical care to identify patients at risk for inadequate pain management and to tailor interventions accordingly. Further research is warranted to better understand the mechanisms through which psychological factors, such as anxiety and depression, influence postoperative pain experiences and treatment adequacy. A deeper understanding of these relationships could inform development of targeted psychological interventions and more individualized pain management strategies.

In this study, the vast majority of participants were male (80.7%), which is consistent with previous reports indicating that men are more frequently affected by traumatic injuries in LMICs due to occupational hazards and greater exposure to mobility-related risks [42, 43]. Although sex was not significantly associated with pain management adequacy in the multivariate models, it is important to acknowledge that gender differences in pain perception, reporting, and treatment responses have been documented in the literature. Studies suggest that women tend to report higher pain intensity and are more likely to receive analgesics, including opioids, compared to men [44–46]. However, due to the limited number of female participants in the present study, our study is underpowered to explore sex-specific differences in pain management outcomes. Future research with more balanced gender representation is warranted to investigate potential sex disparities in analgesic practices and outcomes.

The findings of this study have several important clinical implications. First, the high prevalence of inadequate pain management, particularly in the preoperative phase, shows the need for standardized pain management protocols in fracture care. Such protocols should include timely pain assessment, use of pain catheter, appropriate analgesic selection based on pain severity, and adherence to WHO guidelines. Second, the significant associations between psychological factors and inadequate postoperative pain management support the integration of routine psychological screening into preoperative evaluations. Early identification of patients with elevated anxiety or depressive symptoms may allow for targeted interventions to improve pain outcomes. Third, the strong association between lower educational attainment and inadequate pain management underscores the importance of patient-centered educational strategies. Tailored communication approaches and health literacy-sensitive materials could empower patients to better understand and report their pain, thereby improving the quality of care. Implementing these strategies in resource-limited settings may improve both the effectiveness and equity of perioperative pain management.

### Strengths and Limitations

This study has several strengths. The use of established metrics and validated instruments, such as the PMI and standardized pain assessment tools, enhances the validity and reliability of our findings. The prospective observational design allowed for real-time data collection, thereby minimizing recall bias and improving the accuracy of reported pain experiences and analgesic use. Several limitations should be considered when interpreting our results. First, pain was assessed at only two time points, prior to surgery and 24 h post-surgery, limiting insights into the trajectory of pain from injury through the early recovery period. The short 24-hours follow-up may also fail to capture longer-term postoperative pain management needs. Additionally, since pain was measured only at rest, the findings may not fully capture functional or movement-related pain.

Another limitation is the lack of data on preoperative fracture management and injury severity, which may have influenced preoperative pain levels. The absence of strong opioid use, despite a considerable proportion of patients reporting severe pain, may have affected the assessment of pain management adequacy. This is likely to reflect broader systemic challenges, including limited opioid availability, provider knowledge gaps, and concerns about opioid safety, factors that were not explicitly examined in our study and may act as unmeasured confounders. Finally, the generalizability of the findings may be limited, as the study was conducted in two centers with a relatively small sample size. Future studies with larger, more diverse populations and extended follow-up periods are needed to further validate and expand upon these findings.

## Conclusions

This study highlights a critical gap in perioperative pain management for patients undergoing orthopedic surgery for traumatic fractures in Ethiopia. A substantial proportion of patients received inadequate analgesia, particularly in the preoperative phase. Key factors associated with inadequate pain management included lower educational levels, alcohol use, and elevated anxiety levels.

These findings underscore the importance of addressing social and psychological disparities in pain care. Ensuring equitable pain management for patients with lower educational backgrounds requires targeted interventions that promote health literacy and patient engagement. Moreover, the significant role of psychological factors calls for the integration of routine mental health screening into preoperative assessments. A multifaceted approach combining provider education, structured pain management protocols, and individualized care strategies that account for sociodemographic and psychological factors is essential to improving pain outcomes. By enhancing awareness and clinical training among healthcare providers, and implementing evidence-based guidelines, the quality of perioperative care for this vulnerable patient population can be significantly improved.

## Data Availability

The datasets used and/or analyzed on this study are available from the first author (Mestawet Getachew) on reasonable request.

## Abbreviations

*AaBET*: Addis Ababa Burn Emergency and Trauma
*AOR*: Adjusted Odds Ratio
*BPI*: Brief Pain Inventory
*COR*: Crude Odds Ratio
*HADS*: Hospital Anxiety and Depression scale
*JMC*: Jimma Medical Center
*NRS*: Numeric Rating Scale
*NSAIDS*: Non-Steroid Anti-Inflammatory Drugs
*PMI*: Pain Management Index
*PCS*: Pain Catastrophizing Scale
*SPSS*: Statistical Package for Social Science
*WHO*: World Health Organization
